# Pitfalls of rubella serology while on the brink of elimination: evaluation of national data, Belgium, 2017

**DOI:** 10.2807/1560-7917.ES.2021.26.20.2000074

**Published:** 2021-05-20

**Authors:** Sofie Colman, Kris Vernelen, Bernard China, Dorien Van den Bossche, Laura Cornelissen, Marie-Luce Delforge, Marijke Reynders, Mario Berth, Melissa Depypere, Natasja Van Gasse, Sara Vijgen, Jos Van Acker, An Boel, Elizaveta Padalko

**Affiliations:** 1 Clinical Laboratory of Microbiology, OLVZ Aalst, Aalst, Belgium; 2 Quality of Laboratories, Sciensano, Brussels, Belgium; 3 Experts Committee EQA Infectious serology, Quality of Laboratories, Sciensano, Brussels, Belgium; 4 Institute of Tropical Medicine, Antwerp, Belgium; 5 Epidemiology of Infectious Diseases, Sciensano, Brussels, Belgium; 6 University Hospital Erasme, Brussels, Belgium; 7 General Hospital Sint-Jan Bruges, Bruges, Belgium; 8 General Hospital AZ Alma, Eeklo, Belgium; 9 University Hospital Leuven, Leuven, Belgium; 10 Hospital Network Antwerp, Antwerp, Belgium; 11 General Hospital Jessa, Hasselt, Belgium; 12 General Hospital AZ Sint-Lucas, Ghent, Belgium; 13 University Hospital Ghent, Ghent, Belgium

**Keywords:** rubella, serology, epidemiology

## Abstract

**Background:**

In Belgium, rubella serology is frequently requested in women of childbearing age, despite high vaccination coverage and a near-absence of congenital rubella cases. Different test kits are available and should be standardised by an international standard preparation.

**Aim:**

To analyse and compare rubella serology practices in Belgian laboratories.

**Methods:**

As part of the mandatory External Quality Assessment programme for rubella serology in Belgium, the national public health institute, Sciensano, sent a voluntary questionnaire concerning anti-rubella IgM/IgG analyses in women aged 15 to 45 years in 2017 to 130 laboratories.

**Results:**

The questionnaire response rate was 83.8% (109/130). The majority of 169,494 IgG analyses were performed on Roche (55%), Abbott (17%) and Diasorin (13%) analysers. Not all laboratories used the proposed international cut-off of 10 IU/mL. Assumed median seroprevalence ranged from 76.3% with Liaison (Diasorin) to 96.3% with Modular (Roche). Despite very low rubella incidence in Belgium, 93 laboratories performed 85,957 IgM analyses, with 748 positive and 394 grey zone results. The National Reference Centre for Measles, Mumps and Rubella virus and the National Reference Centre for Congenital infections did not confirm any positive rubella cases in 2017.

**Conclusion:**

This retrospective analysis shows that rubella serology results may differ considerably according to the assay used. It is therefore important to use the same test when comparing results or performing follow-up testing. The number of anti-rubella IgM analyses was very high. Incorrect use of IgM for screening women of childbearing age can lead to unwarranted anxiety and overuse of confirmation tests.

## Introduction

Rubella is endemic throughout the world, predominantly as a childhood disease. Rubella virus (RV) infections occur only in humans and are generally mild, but complications of RV infection do exist. Polyarthralgia is the most common complication in adult women, but occasionally more serious sequelae occur, such as encephalitis and other neurological manifestations. However, the primary public health concern of RV infection is its teratogenicity. RV infection in women during the first trimester of pregnancy can induce a spectrum of congenital defects in the newborn, known as congenital rubella syndrome (CRS). The earlier in gestation the maternal infection occurs, the more severe the damage to the foetus can be [1]. The development of vaccines and implementation of vaccination strategies have substantially reduced the incidence of rubella—and, in turn, of CRS—in countries with vaccination programmes [1]. It is therefore alarming that, according to recent seroepidemiological studies from countries with high vaccination coverage, the percentage of women of fertile age who are not protected against rubella is increasing [2-4]. However, despite an apparently increasing number of seronegative individuals, the number of rubella cases did not increase and practically no CRS cases occurred in countries with high vaccination coverage [5].

Rubella vaccination is included in the basic vaccination schedule in Belgium. Since 1985, a standard administration of a first dose of measles-mumps-rubella (MMR-1) at 12 months old was initiated, with a second dose (MMR-2) at 10 to 12 years old. The second dose was administered for the first time in 1995, on the same birth cohort. Prior to this schedule, a vaccine for rubella was given to 15 year old girls [6]. The seroconversion rate after two doses of measles-mumps-rubella (MMR) approaches 99% and antibodies persist for at least 21 years. With a calculated half-life of 114 years, rubella-specific antibodies may even persist for an entire lifetime [7]. Current vaccination coverage in Belgium for MMR-1 is 95.7%. For MMR-2, the vaccination coverage is 93.4% in Flanders and 75.0% in Wallonia [8]. One dose of the MMR vaccine is considered sufficient to convey immunity to rubella (in contrast to measles) and community immunity can be expected from vaccination coverage of 85–87% [9]. Belgian guidelines recommend testing for anti-rubella IgG during antenatal care only if the individual’s immune status is not known [10].

There are different test systems on the market to determine an individual’s serostatus for rubella [11] and an international standard preparation is available, which should render assay systems comparable. Since the 1980s, anti-rubella IgG assays have been calibrated against the World Health Organization (WHO) International Standard (RUBI-1–94) and the test results are reported in international units per millilitre (IU/mL). Initially, the cut-off for anti-rubella IgG assays was set at 15 IU/mL. However, in the mid-1990s, the Rubella Subcommittee of the National Committee for Clinical Laboratory Standards (CLSI) proposed lowering the breakpoint for rubella immunity from 15 to 10 IU/mL [12]. This recommendation stems from epidemiologic studies, anecdotal reports and the already widespread use of the lower limit in the United States (US), without apparent adverse effects [13].

We investigated which cut-offs for anti-rubella IgG assays are currently used in the different Belgian laboratories. Another aim of this study was to assess national data on the requests and results of anti-rubella IgM/IgG analyses in 2017, in women between 15 and 45 years old, from Belgian laboratories using different kits from different manufacturers.

## Methods

### Study design

A voluntary, retrospective questionnaire was sent out by Quality of Laboratories—a department of Sciensano, the Belgian national public health institute—to all 130 laboratories that perform rubella serology in Belgium. The questionnaire was part of the mandatory External Quality Assessment programme for rubella serology in Belgium. The questionnaire applied to all anti-rubella IgM/IgG analyses in women aged 15 to 45 years, performed in Belgian laboratories in 2017.

In Belgium, an acute rubella case is defined by positive anti-rubella IgM, suspicious clinical presentation and a diagnostically significant titre change in IgG antibody levels between acute and convalescent sera, or documented seroconversion [14].

### Data collection

Data were collected on: (i) which kit was used; (ii) the cut-offs used for positive, grey zone and negative results; (iii) the number of analyses performed; (iv) the number of positive, grey zone and negative results; (v) the number of referrals to a reference centre or another centre and (vi) the number of confirmed rubella cases (Questionnaire provided in Supplement S1). As stated in the survey, a cut-off of 9.9 IU/mL was considered as equal to 10 IU/mL, and 10.9 IU/mL as equal to 11 IU/mL.

The data was anonymised in such a way that the lead investigator could not determine the identity of the laboratories. Only one of the authors had the key for identifying the laboratories, but it was never consulted. Eleven different automated immunoassays for anti-rubella IgG and nine different automated immunoassays for anti-rubella IgM were used in the laboratories. (Supplement S2).

### Statistical analysis

The laboratory cut-offs that were used were compared with those recommended by WHO and the manufacturer, and the serology requests and results for IgM/IgG tests were analysed. Descriptive statistics and data analysis were performed using MedCalc (version 11.6.1.0; MedCalc, Ostend, Belgium).

## Results

The response rate to the questionnaire was 83.8% (109/130).

### Cut-off for rubella immunity

**Figure 1:**
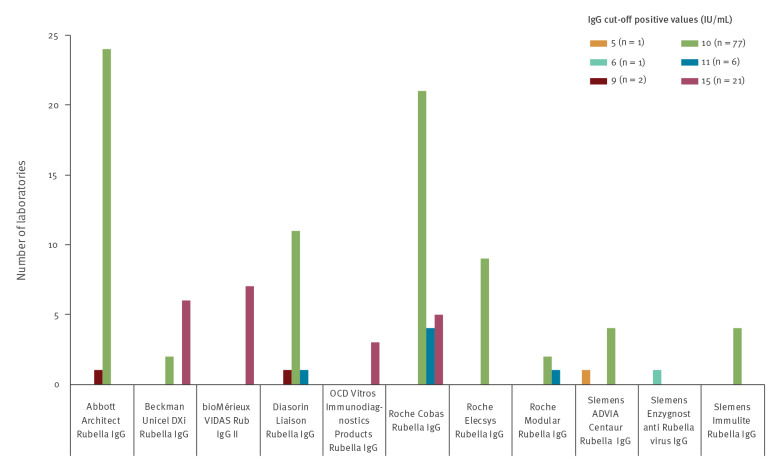
Overview of the anti-rubella IgG cut-offs used to determine positive values, by kit used and by laboratory, Belgium, 2017

Of the 109 participating laboratories, 108 reported the cut-off used to determine positivity. Not all laboratories used the WHO proposed cut-off of 10 IU/mL for anti-rubella IgG. The cut-offs ranged from 5–15 IU/mL. Some manufacturers recommended the use of a grey zone, which was not done by all users of these assays. Eighty-eight laboratories complied with manufacturer recommendations and 63 laboratories used a grey zone in reporting the results. Three of the manufacturers still recommended a cut-off of 15 IU/ml. (Figure 1 and Table 1)

OCD: Ortho Clinical Diagnostics.

Of the 108 participating laboratories, 77 (71%) used the World Health Organization proposed cut-off of 10 IU/mL for anti-rubella IgG.

**Table 1:** Distribution of the anti-rubella IgG cut-offs used for negative, grey zone and positive results, by test kit and manufacturer, Belgium, 2017

Test kit and manufacturer	Number of laboratories using the test	Number of laboratories using the indicated cut-off	Cut-off used (IU/mL)		
			Negative	Grey zone	Positive
Architect Rubella IgG (Abbott, Chicago, Illinois, US)	25	23	< 5	5–10	> 10
		2	< 10	NA	≥ 10
	Manufacturer instructions		< 5	5–9.9	≥ 10
Unicel DXi Rubella IgG (Beckman, Brea, California, US)	8	1	< 5	5–10	> 10
		4	< 10	10–15	> 15
		1	< 10	NA	> 10
		2	< 15	NA	> 15
	Manufacturer instructions		< 10	10–15	> 15
VIDAS Rub IgG II (bioMérieux, Marcy-l'Étoile, France)	7	7	< 10	10–15	> 15
Liaison Rubella IgG (Diasorin, Saluggia, Italy)	13	1	< 5	5–9	> 9
		3	< 5	5–10	> 10
		6	< 7	7–10	> 10
		2	< 10	NA	> 10
		1	< 11	NA	> 11
	Manufacturer instructions		< 7	7–10	> 10
Vitros Immunodiagnostics Products Rubella IgG (Ortho Clinical Diagnostics, Raritan, New Jersey, US)	3	3	< 10	10–15	> 15
Cobas Rubella IgG (Roche, Basel, Switzerland)	30	2	< 7.5	7.5–15	> 15
		1	< 8	8–10	> 10
		1	< 8	8–15	> 15
		4	< 9	9–11	> 11
		20	< 10	NA	> 10
		2	< 10	10–15	> 15
	Manufacturer instructions		< 10	NA	> 10
Elecsys Rubella IgG (Roche)	9	9	< 10	NA	> 10
Modular Rubella IgG (Roche)	3	1	< 9	9–11	> 11
		2	< 10	NA	> 10
	Manufacturer instructions		< 10	NA	> 10
ADVIA Centaur Rubella IgG (Siemens, München, Germany)	5	1	< 5	NA	> 5
		3	< 5	5–10	> 10
		1	< 6	6–10	> 10
	Manufacturer instructions		< 5	5–9.9	≥ 10
Enzygnost Anti-Rubella Virus IgG (Siemens)	1	1	< 4	4–6	> 6
Immulite Rubella IgG (Siemens)	4	4	< 5	5–10	> 10

IU: international units; NA: not applicable; US: United States.

Manufacturers’ instructions were taken from each assay’s package insert.

### Serology results

#### IgG

Of the 109 participating laboratories, 104 reported the number of IgG analyses performed and 103 reported the number of positive, grey zone and negative IgG results. In total, 169,494 IgG analyses were performed, with a median of 1,657 analyses per laboratory (range: 27–18,859) (Table 2). Most IgG analyses were performed on Roche (Basel, Switzerland) (55%), Abbott (Chicago, US) (17%) and Diasorin (Saluggia, Italy) (13%) analysers.

**Table 2:** Number of anti-rubella IgG and IgM analyses performed, by manufacturer and kit, Belgium, 2017 (n = 255,451)

IgG		IgM	
Manufacturer and kit	Number of analyses	Manufacturer and kit	Number of analyses
**Abbott** (Chicago, Illinois, US)	**27,978**	**Abbott**	**13,571**
Architect Rubella IgG		Architect Rubella IgM	
**Beckman (**Brea, California, US)	**7,933**	**Beckman**	**5,083**
Unicel DXi Rubella IgG		Unicel DXi Rubella IgM	2,868
		Access Rubella IgM	2,215
**bioMérieux** (Marcy-l'Étoile, France)	**2,132**	**bioMérieux**	**1,736**
VIDAS Rub IgG II		VIDAS Rub IgM	
**Diasorin** (Saluggia, Italy)	**22,024**	**Diasorin**	**10,716**
Liaison Rubella IgG		Liaison Rubella IgM	
**OCD** (Raritan, New Jersey, US)	**1,116**	**OCD**	NA
Vitros Immunodiagnostics Products Rubella IgG		None^a^	
**Roche** (Basel, Switzerland)	**92,960**	**Roche**	**45,789**
Cobas Rubella IgG	63,026	Cobas Rubella IgM	26,900
Elecsys Rubella IgG	23,590	Modular Rubella IgM	18,889
Modular Rubella IgG	6,344		
**Siemens** (München, Germany)	**15,351**	**Siemens**	**7,558**
ADVIA Centaur Rubella IgG	9,332	Enzygnost Anti-Rubella Virus IgM	48
Enzygnost Anti-Rubella Virus IgG	27		
Immulite Rubella IgG	5,992	Immulite Rubella IgM	7,510
**TOTAL**	**169,494**	**TOTAL**	**85,957**

NA: not applicable; OCD: Ortho Clinical Diagnostics; US: United States.

^a^ No laboratory in Belgium used the OCD kit for anti-rubella IgM in 2017.

**Figure 2:**
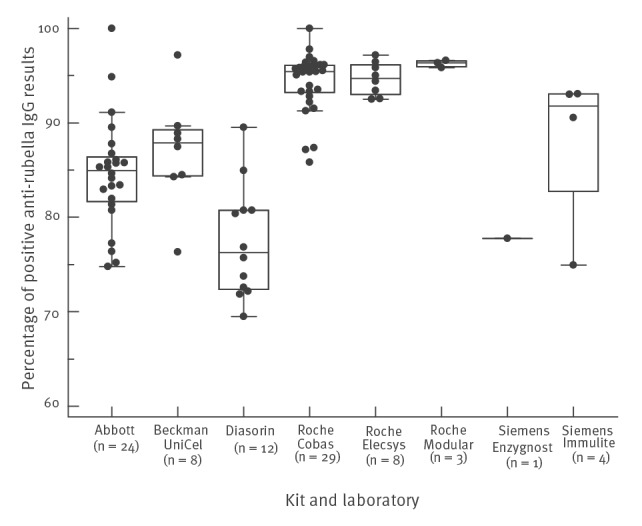
Boxplots of the percentage of positive anti-rubella IgG results, by kit and laboratory, Belgium, 2017

Retrospective analysis of Belgian national data shows large differences in median seropositivity results obtained for women of childbearing age for all laboratories, depending on the assay used. The assumed median seroprevalence in this population group ranges from 76.3% with Liaison (Diasorin) to 96.3% with Modular (Roche) (Figure 2). Numbers are based on the cut-offs used by each individual laboratory.

Each dot represents the result of a separate laboratory. Note that not all participating laboratories responded to all questions in the questionnaire.

**Figure 3:**
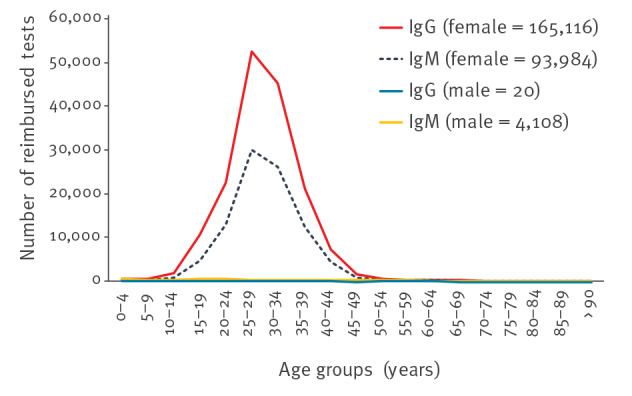
Number of rubella serology tests reimbursed, by age group and sex, Belgium, 2017 (n = 263,228)

Source: Reimbursement data was obtained from the Rijksinstituut voor Ziekte- en Invaliditeitsverzekering/Institut National D'Assurance Maladie-Invalidite (RIZIV/INAMI).

In Belgium, measurement of anti-rubella IgG is performed mostly to establish the immune status of women of childbearing age (Figure 3). In 2017, 96% of all IgG analyses and 92% of all IgM analyses were requested for women aged 15–44 years [15].

#### IgM

**Figure 4:**
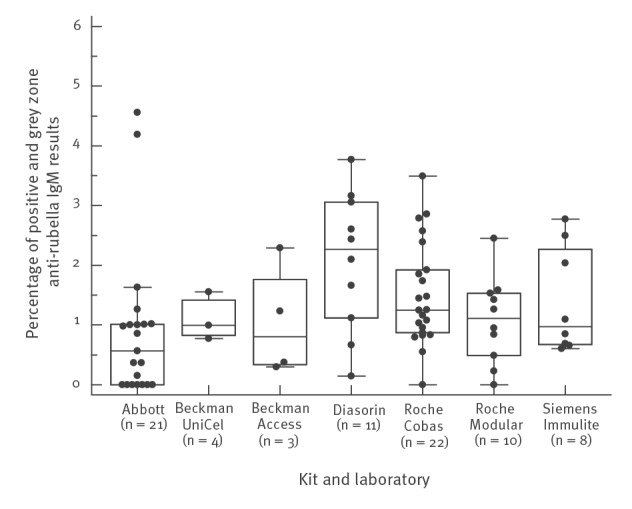
Boxplots of the percentage of positive and grey zone anti-rubella IgM results, by kit and laboratory, Belgium, 2017

Of the 109 participating laboratories, 93 reported the number of anti-rubella IgM analyses performed and 92 reported the number of positive, grey zone and negative IgM results. In total, 85,957 IgM analyses were performed. The median number of analyses performed per laboratory was 527 (range: 7–7,776). Of these analyses, 748 (0.9%) were positive and 394 (0.5%) obtained grey zone results (Figure 4). The Enzygnost kit, which is only used by one laboratory, shows as many as 9 of 48 positive results and 10 of 48 grey zone results. Two laboratories outsourced all IgM analyses. The National Reference Centre (NRC) for Measles, Mumps and Rubella virus performed 50 IgM analyses, of which 28 were clinically suspected cases. However, the NRC did not confirm a single positive case in 2017. The NRC for Congenital Infections, which analysed 106 additional samples during the same year, also did not confirm a single sample as positive for recent infection.

The positive and grey zone results from the Enzygnost kit (Siemens; 19/48) are not displayed.

Each dot represents the results of a separate laboratory. Note that not all participating laboratories responded to all questions in the questionnaire.

## Discussion

Not all Belgian laboratories used the WHO proposed cut-off of 10 IU/mL for anti-rubella IgG results, nor did they all use the cut-off indicated in the manufacturers’ instructions for each assay. Variability in the cut-offs used may play a role in the variability of the qualitative IgG results reported. Some laboratories even used a cut-off lower than the proposed 10 IU/mL. The influence of the cut-off value itself on the qualitative result could not be evaluated, because of the lack of individual test data.

Despite the Belgian recommendation to only test anti-rubella IgG during antenatal care if the immune status is not known, as well as the country’s high vaccination coverage and extremely low rubella prevalence, the number of anti-rubella IgG analyses performed in Belgium was very high. On 1 January 2017, there were 2,184,508 women in Belgium aged 15–45 years; in 2017, there were 119,102 live births and 169,494 anti-rubella IgG tests performed. A thorough prenatal anamnesis could probably reduce the number of routinely performed anti-rubella IgG tests.

To adequately detect high-risk patients and avoid unnecessary revaccination and anxiety during pregnancy, an appropriate definition of seropositivity is essential. Since the available anti-rubella IgG assays use different antigens (total virus, recombinant antigens) and different assay constructions (the conjugate, the assay format (indirect, sandwich, competition, capture)), some differences are to be expected. The intended standardisation (with the international WHO RUBI-1–94 standard preparation) has not been successful, as illustrated by a recent study by Huzly et al. [11], in which they compared 150 clinical samples with 14 anti-rubella IgG immunoassays. Their results showed that up to one third of samples with low anti-rubella IgG tested negative in current IgG assays and the concordance of qualitative results of the 14 anti-rubella-IgG assays was only 56% [11].

In our study, we contribute to these observations by showing that there is a wide range in apparent sensitivity of diagnostic assays measuring anti-rubella IgG in a retrospective analysis that uses real-world national data from Belgium. Median seropositivity for all laboratories, which reflects the overall seroprevalence in Belgium, ranges from 76.3% to 96.3%, depending on the assay used. We can presume that defining rubella immunity by measurement of anti-rubella IgG with some of the current assays can lead to false negative results, potentially triggering unnecessary booster vaccination.

This was apparent in the study of Bouthry et al. They selected a panel of 322 sera collected from pregnant women that tested negative or equivocal for anti-rubella IgG in routine screening. This panel was tested with two reference tests, immunoblotting and neutralisation, and with eight commercial immunoassays widely used in Europe. Their results showed that 57% of the women considered susceptible to RV at prenatal screening were in fact RV seropositive according to both reference tests. As the reference tests provided strong evidence of previous exposure to RV—most likely due to vaccination—their immune status might have needed to be evaluated differently. Certain clinical decisions (e.g. revaccination) might have been unnecessary for these women [16]. Other studies have also shown that in the absence of widespread RV circulation, antibody concentrations will get lower over time and the increasing number of seronegative individuals could just reflect a low negative predictive value of anti-rubella IgG assays [3,17,18]. Similar effects have been shown for measles and varicella-zoster virus, where routine antibody assays have a low sensitivity in highly vaccinated populations [19].

Furthermore, follow-up seroprevalence studies in the same population should be performed using the same kit. When conclusions are drawn from previously published seroprevalence studies, attention should be given to the kit that has been used to conduct the assays.

Dimech et al. conducted an investigation into the lack of standardisation of rubella virus IgG assays. They concluded that the current WHO international standard (RUBI-1–94) fails by three key metrological principles. The standard is not a pure analyte, but is composed of pooled human immunoglobulin [20]. However, anti-rubella IgG response is polyclonal and antibodies change as the immune response matures. Therefore, assays are detecting and quantifying multiple different antibodies in vaccinated populations, whereas the international standard is a pooled polyclonal antibody preparation developed when vaccination coverage was much lower [21]. Second, the reference methods used to assign a value to RUBI-1–94 could not be considered reference procedures. The value was assigned by testing with haemagglutination inhibition, radial haemolysis and enzyme immunoassays that have since been superseded by arguably superior technology. Finally, no measurement uncertainty estimations have been provided [20].

Even though a report expressed in IU/mL implies comparability from one assay to another, our and previous investigations indicate that the assumption of transferability of IU/mL is incorrect. There clearly is a need for further harmonisation. Within the clinical diagnostic community, some experts propose that clinically validated, robust qualitative tests be introduced to replace quantitative assays. Immunoblot-detecting antibodies to rubella-specific E1 antigens might be considered a potential reference method for confirmation of protective immunity [11].

New recommendations about RV serology were drafted as a result of the WHO consultation on 30 June 2017. These were subsequently discussed at the meeting of WHO-Expert Committee on Biological Standardization (WHO-ECBS) in October 2017. WHO-ECBS agreed that RUBI-1–94 should continue to be made available as a well characterised reference material. Manufacturers, regulators and assay users should be made aware of this lack of commutability and other limitations, and this information should be included in the manufacturers’ instructions. In addition, those involved in diagnostics—e.g. diagnostic expert committees, vaccine efficacy evaluators and regulators—should be encouraged to reconsider the appropriateness of quantitative anti-rubella measurement for the determination of immune status and the use of 10 IU/mL as a cut-off point for assessing immune protection [22].

In countries that have achieved or are approaching rubella elimination, the positive predictive value (PPV) of IgM serology decreases as more false positives are obtained. Thorough case investigations, including high-quality laboratory testing, are crucial. Furthermore, it is important to use all available information including clinical, epidemiological and patient data. In high-incidence settings, rubella diagnoses can be made based on clinical data. However, in low-incidence regions and settings where elimination has been reached, diagnosis of rubella infections requires confirmation using accurate laboratory testing. For rubella, IgM antibodies appear within 3–4 days after rash onset and are sometimes detectable up to 2 months after illness. In Ontario, Canada, where rubella is eliminated, all anti-rubella IgM testing is performed at public health laboratories and public health investigations are recorded in a provincial database. Analysis of these data concluded that the PPV for IgM rubella testing was only 3.6% for the period 2009 to 2014, with only five confirmed cases even though 10,220 laboratory analyses were performed. When there is a low prevalence of disease, a positive result is most likely due to a false reactivity, rather than a true infection [23].

Rubella was declared eliminated in the US in 2004 [24]. However, imported cases from countries where rubella is endemic still occur. The New York City Department of Health and Mental Hygiene reviewed all 199 positive rubella reports from 2012 to 2013, including 188 (95%) first reported by laboratories based on positive IgM results. Of all of these reports, 77.9% were tested for rubella IgM when there was no clinical suspicion of rubella disease, 19.6% were tested for diagnostic purposes and 2% had an unknown test purpose. Only two cases were confirmed, so the PPV was very low, supporting the results of the Ontario study. The New York study concluded that limiting rubella testing to patients with a clinical suspicion of infection has the potential to reduce false-positive rubella IgM results [25].

In our retrospective analysis, the number of anti-rubella IgM analyses performed was very high: 85,957. The enormous number of IgM analyses generates a cost to the National Institute for Health and Disability Insurance of ca €200,000/year, the equivalent of 7,600 consultations [15]. A total of 748 tests returned a positive result for anti-rubella IgM, despite the very low prevalence of acute rubella. The NRCs did not confirm any positive rubella cases for 2017. There have been no congenital rubella cases reported in Belgium for the past 10 years, with the exception of one imported case in 2012 [6]. False-positive anti-rubella IgM results can cause unnecessary stress during pregnancy. Our results show that positive IgM results should be interpreted with caution in Belgium in women of childbearing age and are requested far too often. Furthermore, data from the survey indicated that there were six cases of acute rubella infection. Upon further inquiry, these diagnoses of acute infection seemed to rely solely on the confirmation of positive IgM tests in another laboratory, and no information on clinical symptoms was available; therefore, the cases did not meet the case definition criteria. In general, we recommend not to systematically test for IgM antibodies in low prevalence areas.

Our study has some limitations. The questionnaire was voluntary, which could imply a selection bias of the laboratories. In contrast to the national health insurance data from Rijksinstituut voor Ziekte- en Invaliditeitsverzekering/Institut National D'Assurance Maladie-Invalidite (RIZIV/INAMI), we cannot provide the exact number of analyses performed from the questionnaire. Since some samples were sent to the NRCs or another laboratory, a small number of patient results are duplicated. However, the number of referred, duplicate samples is negligible compared with the total number of performed analyses. With a response rate of almost 84%, we consider our data representative of the Belgian clinical diagnostic laboratory landscape. We could not perform a sensitivity analysis of the results since we did not receive the individual measurement data from each laboratory. Therefore, our data provide only indirect estimates of the sensitivity of the different assays. The use of different cut-offs could also lead to differences in interpretation of serological status. As Figure 1 shows, Diasorin users don’t use a higher cut-off, so this alone cannot be the explanation of the lower seropositivity. Finally, since the data were anonymously evaluated, and we have no data on the exact population distribution and characteristics, possible differences between regions and laboratories might have effects on the seropositivity rate from different laboratories.

Belgium has committed to achieving the WHO measles and rubella elimination goal by 2020 [26]. One of the core strategies to reach this goal is to monitor the disease using effective surveillance and to evaluate programmatic efforts to ensure progress. To optimise surveillance in Belgium, laboratories are encouraged to send samples from patients with suspected rubella virus infection to the NRC for avidity analysis and PCR. Awareness campaigns targeting gynaecologists and general practitioners encourage clinicians to only request anti-rubella IgG to determine serostatus in women of reproductive age. Our study indicates that these additional measures are necessary to promote responsible prescription of tests.

## Conclusion

This retrospective analysis of Belgian data from 2017 suggests a wide variety in the sensitivity of diagnostic assays measuring anti-rubella IgG in women aged 15–45 years. Median estimated seroprevalence varies strongly depending on the assay used, despite current vaccination coverage of 95.7% for MMR-1. Furthermore, the number of anti-rubella IgM and IgG analyses was very high, especially considering that testing for anti-rubella IgG is only recommended if an individual’s immune status is not known. Not all Belgian laboratories used the WHO proposed cut-off of 10 IU/mL for anti-rubella IgG results, nor did they all use the cut-off indicated in the manufacturers’ instructions for each assay. Neither NRC confirmed any positive rubella cases for 2017. The incorrect use of IgM as a screening tool in women of childbearing age can lead to unwarranted anxiety, as well as overuse of foetal imaging and confirmation tests.

## Supplementary Data

187000 Supplementary Material
