# Supplementary material for: Pitfalls of rubella serology while on the brink of elimination: evaluation of national data, Belgium, 2017
Source: Euro Surveill. 2021 May 20;26(20):2000074. doi: 10.2807/1560-7917.ES.2021.26.20.2000074 (PMC8138961; doi:10.2807/1560-7917.ES.2021.26.20.2000074)
Supplement: Supplementary Material [file 20-00074_COLMAN_Supplement.pdf]

This supplementary material is hosted by Eurosurveillance as supporting information alongside the article **Pitfalls of rubella serology while on the brink of elimination: evaluation of national data, Belgium, 2017** on behalf of the authors who remain responsible for the accuracy and appropriateness of the content. The same standards for ethics, copyright, attributions and permissions as for the article apply. Supplements are not edited by *Eurosurveillance* and the journal is not responsible for the maintenance of any links or email addresses provided therein.

Supplement S1: Questionnaire

1. Which method did you use in routine in 2017 for determination of

Anti-rubella IgG	Manufacturer: ……..…..	Kit: ……..…….
Anti-rubella IgM	Manufacturer: ……..…..	Kit: …………..….
Anti-rubella IgG avidity	Manufacturer: ……..…..	Kit: …………...

2. Which cut-off (in IU/mL) was used in 2017 with these methods for determination of anti-rubella IgG and anti-rubella IgM in your laboratory?
   a. IgG

Negative: < ……..
Grey zone:
Positive: > ……..

   b. IgM

Negative: < ……..
Grey zone:
Positive: > ……..

3. Number of anti-rubella IgG analyses in women 15-45 years in 2017: …………………….

   a. Number of negative anti-rubella IgG results in women 15-45 years in 2017: …………………….
   b. Number of grey zone anti-rubella IgG results in women 15-45 years in 2017: …………………….
   c. Number of positive anti-rubella IgG results in women 15-45 years in 2017: …………………….

4. Number of anti-rubella IgM analyses in women 15-45 years in 2017: …………………….

   a. Number of negative anti-rubella IgM results in women 15-45 years in 2017: …………………….
   b. Number of grey zone anti-rubella IgM results in women 15-45 years in 2017: …………………….
   c. Number of positive anti-rubella IgM results in women 15-45 years in 2017: …………………….

5. Does your laboratory send samples to one of the national reference centres (NRC) or another laboratory?

❒ NRC Measles, Mumps, Rubella

❒ NRC congenital infections

This supplementary material is hosted by Eurosurveillance as supporting information alongside the article **Pitfalls of rubella serology while on the brink of elimination: evaluation of national data, Belgium, 2017** on behalf of the authors who remain responsible for the accuracy and appropriateness of the content. The same standards for ethics, copyright, attributions and permissions as for the article apply. Supplements are not edited by *Eurosurveillance* and the journal is not responsible for the maintenance of any links or email addresses provided therein.

❒ Other laboratory

❒ No

a. Number of samples in 2017: …………………….

b. Which samples?

❒ Clinically suspected
❒ Not-negative anti-rubella IgM
❒ Selected samples

c. If preselection, which criterium:
……………………………………….…………………

6. Did you have confirmed cases of acute rubella infection in 2017 in your laboratory?

❒ Yes ❒ No
Number: ……………….
How did the confirmation happen? (After exclusion recent vaccination)
❒ IgM positive and significant titer rise in IgG on paired samples (n=…………)
❒ IgM positive and low anti-rubella IgG avidity (n=…………)
❒ Molecular diagnostics (PCR) positive (n=…………)
❒ Other criteria (n=…………)
Specify: ……………………………………………………

This supplementary material is hosted by Eurosurveillance as supporting information alongside the article **Pitfalls of rubella serology while on the brink of elimination: evaluation of national data, Belgium, 2017** on behalf of the authors who remain responsible for the accuracy and appropriateness of the content. The same standards for ethics, copyright, attributions and permissions as for the article apply. Supplements are not edited by *Eurosurveillance* and the journal is not responsible for the maintenance of any links or email addresses provided therein.

Supplement S2: Overview of automated immunoassays for anti-rubella IgG and anti-rubella IgM

Eleven different automated immunoassays for anti-rubella IgG were used in the laboratories in 2017.

Kit	Instrument	Manufacturer
Architect Rubella IgG	Abbott Architect system	Abbott, Chicago, Illinois, US
Unicel DXi Rubella IgG	Beckman Coulter system	Beckman Coulter, Brea, California, US
VIDAS RUB IgG II	mini VIDAS System	bioMérieux, Marcy-l'Étoile, France
DiaSorin Liaison Rubella IgG	LIAISON system	Diasorin, Saluggia, Italy
Vitros Rubella IgG	Vitros ECi System	Ortho Clinical Diagnostics, Raritan, US
Cobas Rubella IgG	Roche ELECSYS system	Roche, Basel, Germany
Elecsys Rubella IgG	Roche ELECSYS system	Roche, Basel, Germany
Modular Rubella IgG	Roche Modular system	Roche, Basel, Germany
ADVIA Centaur Rubella G	Siemens ADVIA Centaur system	Siemens Healthcare, München, Germany
Enzygnost Rubella IgG	Siemens system	Siemens Healthcare, München, Germany
Immulite Rubella IgG	Siemens system	Siemens Healthcare, München, Germany

Table 1: Overview of automated immunoassays for anti-rubella IgG.

Nine different automated immunoassays for anti-rubella IgM were used in the laboratories in 2017.

Kit	Instrument	Manufacturer
Cobas Rubella IgM	Roche ELECSYS system	Roche, Basel, Germany
Modular Rubella IgM	Roche Modular system	Roche, Basel, Germany

This supplementary material is hosted by Eurosurveillance as supporting information alongside the article **Pitfalls of rubella serology while on the brink of elimination: evaluation of national data, Belgium, 2017** on behalf of the authors who remain responsible for the accuracy and appropriateness of the content. The same standards for ethics, copyright, attributions and permissions as for the article apply. Supplements are not edited by *Eurosurveillance* and the journal is not responsible for the maintenance of any links or email addresses provided therein.

Architect Rubella IgM	Abbott Architect system	Abbott, Chicago, Illinois, US
DiaSorin Liaison Rubella IgM	LIAISON system	Diasorin, Saluggia, Italy
Enzygnost Rubella IgM	Siemens system	Siemens Healthcare, München, Germany
Immulite Rubella IgM	Siemens system	Siemens Healthcare, München, Germany
Unicel DXi Rubella IgM	Beckman Coulter system	Beckman Coulter, Brea, California, US
Access Rubella IgM	Beckman Coulter system	Beckman Coulter, Brea, California, US
VIDAS RUB IgM	mini VIDAS System	bioMérieux, Marcy-l'Étoile, France

Table 2: Overview of automated immunoassays for anti-rubella IgM.
